# Why Andes hantavirus is not the next SARS-CoV-2: contrasting viral shedding, transmissibility and genomic patterns

**DOI:** 10.2807/1560-7917.ES.2026.31.22.2600428

**Published:** 2026-06-04

**Authors:** Antonin Bal, Virginie Sauvage, Maude Bouscambert-Duchamp, Bruno Lina, Alexandre Gaymard, Jean-Claude Manuguerra

**Affiliations:** 1 Hospices Civils de Lyon (HCL), Centre National de Référence des virus des infections respiratoires, Institut des Agents Infectieux, Laboratoire de Virologie, Lyon, France; 2 Centre International de Recherche en Infectiologie (CIRI), Team CARVI, Univ Lyon, Inserm, U1111, Université Claude Bernard Lyon 1, Lyon, France; 3 Institut Pasteur, Université Paris Cité, Environment and Infectious Risk Research Unit, French National Reference Centre for Hantaviruses, Paris, France; 4 Institut Pasteur, Université Paris Cité, Environment and Infectious Risk Research Unit, Laboratory for Urgent Response to Biological Threats (CIBU), Paris, France

**Keywords:** SARS-CoV-2, Hantavirus, Viral Shedding, Transmissibility, Diagnostic

## Abstract

A cruise ship-associated Andes hantavirus outbreak has raised questions usually associated with respiratory viruses, including transmissibility and pandemic risk. Although Andes virus may enter through the respiratory route, cause severe respiratory disease and under close contact spread between humans, it differs fundamentally from SARS-CoV-2. The ecology is rodent-borne, pathogenesis is vascular, diagnosis is centred on blood PCR and serology, and genetic diversity is mainly shaped by reservoir ecology and geography rather than by sustained human-to-human transmission and immune selection.

In May 2026, multi-country cases of Andes hantavirus infection linked to the MV Hondius cruise ship sparked global concern because of cases presenting severe respiratory disease, prompting comparisons with the early COVID-19 pandemic [1]. We compared Andes hantavirus with SARS-CoV-2 to clarify the main virological differences that support current assessments by the World Health Organization (WHO) and European Centre for Disease Prevention and Control (ECDC) of low to very low risk to the general population, while addressing public concerns raised by a severe respiratory outbreak in a closed setting [1,2].

## Different viral receptors

Andes virus, classified as *Orthohantavirus andesense*, is a New World orthohantavirus species. The primary reservoir of the virus is *Oligoryzomys longicaudatus*, long-tailed pygmy rice rat, a rodent endemic in Argentina and Chile and absent in Europe [3]. Human infection usually follows inhalation of aerosols or dust contaminated by rodent urine, faeces or saliva. New World hantaviruses use protocadherin-1 (PCDH1) as a key entry factor that is mainly expressed in pulmonary endothelial cells [4]. In addition, Andes virus infection of non-ciliated cells in primary human airway epithelial cultures, with bidirectional virus secretion, has been reported, possibly involving β3-integrins [5]. This observation may contribute to the biological plausibility of human-to-human transmission through respiratory or oro-respiratory secretions, while not implying efficient SARS-CoV-2-like respiratory spread. In SARS-CoV-2 infection, ACE2 receptor and TMRSS2 protease are enriched in nasal and respiratory epithelial compartments, supporting efficient replication and shedding of the virus from the upper and lower respiratory tract [6]. Severe Andes virus disease, however, is mainly driven by endothelial dysfunction, capillary leak, pulmonary oedema, hypoxaemia and shock [7], rather than by primary epithelial respiratory replication and the inflammatory response seen in severe COVID-19 cases [8].

## Viral shedding

Incubation period for SARS-CoV-2 infection has shortened across variants, from ca 4–5 days for the early lineages to around 3–4 days for Omicron, contrasting with Andes virus, for which incubation can last up to 6 weeks with a median of around 20 days [9-11]. Although Andes virus is primarily detected in blood-derived samples, its presence in saliva and nasopharyngeal samples supports the possibility of close contact transmission through infectious secretions. Detection of Andes virus in respiratory samples reflects several mechanisms: systemic viraemia with seeding of oral compartments, vascular leakage, infected cells in secretions, gingival fluid that may reflect inflammatory exudate containing infected mononuclear cells, including macrophages, or virus derived from systemic viraemia and possible local replication in salivary gland secretory cells [12]. In a prospective Chilean study of 131 patients with acute Andes virus infections, viral RNA was detected in all buffy coat samples during the acute phase, declining to 93% 29 days post-symptom onset. Detection in saliva was less frequent, with a detection in ca 12% during both the acute phase and convalescent phases. Infectious virus was recovered from 18 of 43 PCR-positive acute phase fluids, including saliva, nasopharyngeal secretions and urine [13]. Andes virus RNA was also reported after infection in a convalescent patient; however, infectious virus could not be recovered from semen samples, and the implications for transmission remain uncertain [14]. This pattern contrasts SARS-CoV-2 in which saliva, nasopharyngeal and lower respiratory tract† are the main sites of shedding. For the Omicron variant, which is associated with higher replication in the upper respiratory tract than the previous variants, a systematic review estimated the duration of shedding of a pooled viable virus in the upper respiratory tract of 5.2 days and a mean PCR positivity duration of 10.8 days [15]. The RNA of SARS-CoV-2 has also been detected in blood, cerebrospinal fluid and stool while detection in urine or semen appears uncommon, and infectious virus is rarely isolated from non-respiratory specimens [16,17].

## Transmissibility

In the early phase of the COVID-19 pandemic, the Diamond Princess ship outbreak illustrated the efficient human-to-human respiratory transmission of SARS-CoV-2 in a closed setting. Modelling estimated an initial reproduction number (R_0_)† of 2.9 and a doubling time of 3.4 days, with 712 (19%) infections among 3,711 people mainly reported in the first 3 weeks after the onset of symptom of the index case [18-20]. By contrast, as of 4 June 2026 and 2 months after the first symptoms reported by the index case, 13 (9%) confirmed or probable Andes virus cases were reported among 147 people on MV Hondius [1]. Absence of documented secondary cases outside the vessel at that time should not be interpreted as excluding subsequent secondary transmission given the long incubation period [1,21].

The SARS-CoV-2 Omicron variant, detected in late 2021 and characterised by high receptor-binding-domain evolution, had R_0_ estimates of around 8† and a short doubling time of ca 2 days, highlighting the rapid spread of this variant [22-24]. The 2018–2019 Epuyen outbreak of Andes virus in Argentina is the strongest evidence of person-to-person transmission, with 34 confirmed cases, 11 deaths, epidemiological chains and genomic support for successive human transmission [25]. The median R_0_ was estimated at ca 2.1 before isolation, quarantine and active contact tracing, decreasing below 1 after implementation of control measures [25]. In addition, a non-peer-reviewed modelling study suggested that ca 70% of single-case Andes virus introductions are associated with no secondary cases and 13% with only one secondary case [10]. In Chile, Ferrés et al. followed 476 household contacts of 76 patients with hantavirus cardiopulmonary syndrome. Sixteen contacts developed disease; the risk was highest among sexual partners of index cases, reaching 17.6% compared with 1.2% among other household contacts. There was no evidence of asymptomatic transmission or asymptomatic seroconversion, although Andes virus RNA was detected in peripheral blood 5–15 days before symptom onset or seroconversion in contacts who later developed disease [26].

The role of asymptomatic or pauci-symptomatic Andes virus infection remains a key uncertainty. Seroprevalence studies and contact investigations suggest that some infections may be missed by surveillance focused on severe cardiopulmonary syndrome, and viraemia can precede symptoms and seroconversion [9,26]. Transmission may occur around the prodromal or early symptomatic phase; however, the precise timing of infectiousness remains incompletely defined [10,21,25,26]. This differs from SARS-CoV-2: for Omicron, household transmission modelling estimated that pre-symptomatic transmission was a major driver of spread [27].

## Diagnostic strategies

For COVID-19, PCR from upper respiratory tract samples† or antigen testing is central for diagnosis while the clinical utility of serological testing is limited. In highly immune adult populations, SARS-CoV-2 viral load may peak around the fourth day of symptoms [28]. Antibody responses for SARS-CoV-2 generally occur too late for first-line acute diagnosis; earlier studies showed that IgG and IgM seroconversion usually developed after symptom onset, with IgG detectable in all patients by 19 days in one series [29]. Seroprevalence for SARS-CoV-2† is now very high, which further underlines the limited utility of this approach.

By contrast, the diagnosis of hantavirus infection relies on different approaches according to the clinical syndrome and virus involved. For suspected hantavirus pulmonary syndrome, including Andes virus infection, RT-PCR on blood-derived specimens is particularly important during the acute phase, complemented by serology. In studies mainly conducted on Old World hantaviruses, IgM antibodies are detectable in ca 75% of cases and IgG antibodies in approximately two thirds of cases within 3 days after symptom onset, with both detected in nearly all cases within 7 days. Antibodies of IgM usually decline over 1–2 months, whereas IgG may persist for years, possibly lifelong [9,30]. For molecular detection, buffy coat, whole blood and plasma are the most relevant specimens, whereas saliva testing may contribute to transmission assessment. Molecular detection is most useful early during the prodromal and acute cardiopulmonary phases, with detection rates around 90% in blood compartments within the first 3 days after symptom onset [9,13]. Detection by PCR may precede symptoms and antibody detection in exposed contacts, while IgM and IgG are often detectable by the time patients become symptomatic [13,26].

## Genomic characteristics

The genomic evolution of SARS-CoV-2 is mainly driven by sustained transmission chains and immune selection. Spike mutations such as D614G, which emerged early during the pandemic, increased viral fitness and transmissibility, while later spike mutations contributed to immune escape, altered transmission dynamics and, for some variants such as Delta variant, increased disease severity [31,32].

Andes virus diversity is structured mainly by geography and rodent reservoirs [33] and can show high short-term substitution rates, estimated in the range of 10^−2^ to 10^−4^ substitutions/site/year. In comparison, SARS-CoV-2 has an evolutionary rate of ca 10^−3^ substitutions/site/year, but variants of concern can show episodic acceleration, with rates several fold above the background rate over weeks to months [34]. Preliminary genomic analyses reported very limited diversity among available sequences of the current MV Hondius outbreak that fall within known South American diversity [35,36]. Candidate mutations associated with animal model attenuation or person-to-person transmission have been proposed, but no single mutation, as the D614G for the SARS-CoV-2, should be treated as a deterministic marker of human adaptation, increased transmissibility or virulence [37]. Experimental work has also shown high genomic stability after repeated in vivo passage in Syrian hamsters, suggesting that short transmission chains may occur without rapid accumulation of adaptive mutations [38].

## Conclusion

Andes virus differs fundamentally from SARS-CoV-2: ecology is rodent-borne, pathogenesis is vascular, diagnosis is blood- and serology-centred, and respiratory transmission is limited. Public health responses should combine environmental investigation, isolation of suspected cases, extended contact tracing, without either minimising transmission potential or invoking a SARS-CoV-2-likespread. These conclusions are consistent with the Andes virus risk assessments issued by WHO, ECDC, which have not suggested a SARS-CoV-2-like pandemic risk.

## Data Availability

This manuscript is based on publicly available data and published scientific literature.

## †Author’s correction

At the request of the authors, the following changes were made on 5 June 2026.

In the section entitled ‘Viral shedding’, in the sentence ‘This pattern contrasts SARS-CoV-2 in which saliva, nasopharyngeal and lower respiratory tract samples are the main sites of shedding’, the word ‘samples’ was deleted.

In the section entitled ‘Transmissibility’, the abbreviation R_0_ was added after first mentioning reproduction number. In the sentence ‘The SARS-CoV-2 Omicron variant, detected in late 2021 and characterised by high receptor-binding-domain evolution, had R_0_ estimates of around 8 days and a short doubling time of ca 2 days, highlighting the rapid spread of this variant’, the word ‘days’ was deleted after the number 8.

In the section entitled ‘Diagnostic strategies’, the first sentence was modified from ‘For COVID-19, upper respiratory tract PCR or antigen testing is central for diagnosis while the clinical utility of serological testing is limited’ to ‘For COVID-19, PCR from upper respiratory tract samples or antigen testing is central for diagnosis while the clinical utility of serological testing is limited’. In the sentence: ‘Seroprevalence for COVID-19 is now very high, which further underlines the limited utility of this approach’, the word COVID-19 was changed to SARS-CoV-2.
