# Author’s correction for Euro Surveill. 2026;31(22)

**DOI:** 10.2807/1560-7917.ES.2026.31.23.260611c

**Published:** 2026-06-11

**Authors:** Eurosurveillance editorial team

**Affiliations:** 1 European Centre for Disease Prevention and Control (ECDC), Stockholm, Sweden

In the article ‘Why Andes hantavirus is not the next SARS-CoV-2: contrasting viral shedding, transmissibility and genomic patterns’ by Bal et al., published on 4 June 2026, the following changes were made on 5 June 2026, at the request of the authors.

In the section entitled ‘Viral shedding’, in the sentence ‘This pattern contrasts SARS-CoV-2 in which saliva, nasopharyngeal and lower respiratory tract samples are the main sites of shedding’, the word ‘samples’ was deleted.

In the section entitled ‘Transmissibility’, the abbreviation R_0_ was added after first mentioning reproduction number. In the sentence ‘The SARS-CoV-2 Omicron variant, detected in late 2021 and characterised by high receptor-binding-domain evolution, had R_0_ estimates of around 8 days and a short doubling time of ca 2 days, highlighting the rapid spread of this variant’, the word ‘days’ was deleted after the number 8.

In the section entitled ‘Diagnostic strategies’, the first sentence was modified from ‘For COVID-19, upper respiratory tract PCR or antigen testing is central for diagnosis while the clinical utility of serological testing is limited’ to ‘For COVID-19, PCR from upper respiratory tract samples or antigen testing is central for diagnosis while the clinical utility of serological testing is limited’. In the sentence: ‘Seroprevalence for COVID-19 is now very high, which further underlines the limited utility of this approach’, the word COVID-19 was changed to SARS-CoV-2.

